# Tuning of Kalman Filter Parameters via Genetic Algorithm for State-of-Charge Estimation in Battery Management System

**DOI:** 10.1155/2014/176052

**Published:** 2014-08-05

**Authors:** T. O. Ting, Ka Lok Man, Eng Gee Lim, Mark Leach

**Affiliations:** ^1^Department of Electrical and Electronic Engineering, Xi'an Jiaotong-Liverpool University, No. 111, Ren'ai Road, HET, SIP, Suzhou, Jiangsu 215123, China; ^2^Department of Computer Science and Software Engineering, Xi'an Jiaotong-Liverpool University, No. 111, Ren'ai Road, HET, SIP, Suzhou, Jiangsu 215123, China; ^3^Department of Computer Science, Yonsei University, 50 Yonsei-ro, Seodaemun-gu, Seoul 120-749, Republic of Korea

## Abstract

In this work, a state-space battery model is derived mathematically to estimate the state-of-charge (SoC) of a battery system. Subsequently, Kalman filter (KF) is applied to predict the dynamical behavior of the battery model. Results show an accurate prediction as the accumulated error, in terms of root-mean-square (RMS), is a very small value. From this work, it is found that different sets of **Q** and **R** values (KF's parameters) can be applied for better performance and hence lower RMS error. This is the motivation for the application of a metaheuristic algorithm. Hence, the result is further improved by applying a genetic algorithm (GA) to tune **Q** and **R** parameters of the KF. In an online application, a GA can be applied to obtain the optimal parameters of the KF before its application to a real plant (system). This simply means that the instantaneous response of the KF is not affected by the time consuming GA as this approach is applied only once to obtain the optimal parameters. The relevant workable MATLAB source codes are given in the appendix to ease future work and analysis in this area.

## 1. Introduction

**Figure 1:**
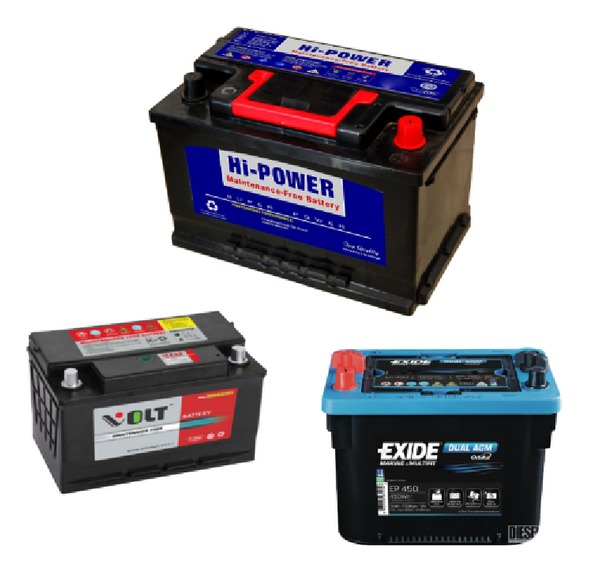
Typical Lithium-ion battery packs for electric vehicle.

Battery Management System (BMS) [1–3] comprises of mechanism that monitors and controls the normal operation of a battery system so as to ensure its safety while maintaining its State-of-Health (SoH). The BMS, in essence, measures the voltage, current, and temperature of each cell in a battery pack. These data are then analyzed by a management system that guarantees safe and reliable operations. A common example of an independent battery pack is portrayed in Figure 1. The battery is an essential component and should be accurately modeled in order to design an efficient management system [4]. Hence, a generic tool to describe the battery performance under a wide variety of conditions and applications is highly desirable [4]. As such, electrical modeling is necessary to provide such a tool that enables visualization of the processes occurring inside rechargeable batteries. These generic models are necessary for the development of battery management algorithms. These algorithms control the operation and maintain the performance of battery packs. In short, the ultimate aim of BMS is to prolong battery life, while ensuring reliable operation alongside many applications, especially in photovoltaic systems [5–7].

Battery modeling is performed in many ways depending on the types of battery. In general, the resulting battery model is a mathematical model comprising numerous mathematical descriptions [8]. Ultimately, battery models aim to determine the state-of-charge (SoC) of the battery system. However, the complexity of the nonlinear electrochemical processes has been a great barrier to modeling this dynamic process accurately. The accurate determination of the SoC will enable utilization of the battery for optimal performance and long-life and prevent irreversible physical damage to the battery [9]. The solution to the SoC via neural networks [10] and fuzzy logic [11] has been difficult and costly for online implementation due to the large computation required, causing the battery pack controller to be heavily loaded. This may however be a good alternative in the near future as the computational power of processing chips increase alongside their declining cost.

**Figure 2:**
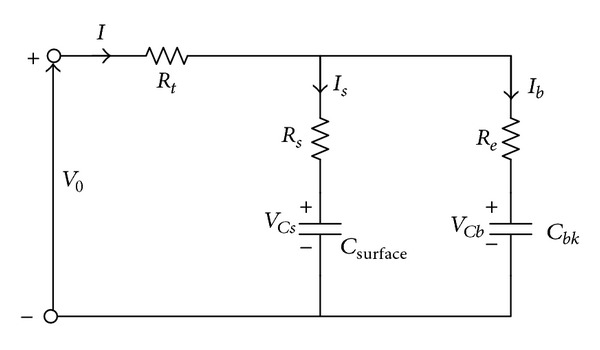
Schematic of RC battery model.

The state-estimation process usually leads to some state variables in a dynamical system. The SoC is a measure of a battery's available power and thus it is important to calculate this value accurately from BMS by the cell voltage, temperature, and polarization effect caused by the electrolyte concentration gradient during high rate charging/discharging cycle [12]. Recently, the battery state-of-charge (SoC) is one of the significant elements attracting significant attention [13, 14]. By definition, SoC is the ratio of remaining capacity to the nominal capacity of the battery. Here, the remaining capacity is the number of ampere-hours (Ah) that can be extracted at normal operating temperature. The mathematical expression for the SoC is given in [13, 14], which is

(1) $$\begin{array}{c} Z\left(t\right)=Z\left(0\right)+\overset{t}{\underset{0}{\int }}\frac{I_{b}\left(\tau \right)}{C_{n}}\text{d}\tau , \end{array}$$

where *t* is time, *z*(*t*) is battery SoC, in amphere-hours (Ah), *I*_*b*_ is current flowing through the battery (passing through *C*_bk_), illustrated in Figure 2, and *C*_*n*_ is nominal battery capacity. For charging, *I*_*b*_ > 0 and for discharging, *I*_*b*_ < 0.

From this mathematical expression, it is noted that the SoC cannot be explicitly measured. In the literature, there is a myriad of methods dealing with predicting and estimating of SoC. The most popular of these methods are described in the following paragraphs.

At present, Coulomb-counting [15], also known as charge counting, or current integration is the most commonly used technique; it requires dynamic measurement of battery current. This is an open-loop method; however, it suffers from a reliance on the mathematical integration, and errors (noise, resolution, and rounding) are cumulative, which can lead to large SoC errors at the end of the integration process in (1). On the positive side, if an accurate current sensor is incorporated, the implementation will be much easier.

Another prominent SoC estimator is the well-known Kalman filter (KF), invented by Kalman in 1960. Although his popular work was published almost 54 years ago in [16], it remains as an important citation source in the literature. Readers who are new to this method can refer to an excellent KF tutorial by Faragher in [17]. The KF method is a well-known technique for dynamic state estimation of systems such as target tracking, navigation, and also battery systems [18, 19]. The state-of-the-art method provides recursive solution to linear filtering for state observation and prediction problems. The key advantage of the KF is that it accurately estimates states affected by external disturbances such as noises governed by Gaussian distribution. On the contrary, the disadvantage of KF is that it requires highly complex mathematical calculations. This can be realized by a state-space model, as shown in previous work by the author in [20, 21]. The modeling is a heavy duty task and is also presented in this work to ease the understanding of readers. As such, there exist some possibilities of divergence due to an inaccurate model and complex calculation. In the case of a slow processor, the calculation results may be delayed and exceed the sampling interval, thereby result in an inaccurate tracking.

Various artificial intelligence (AI) methods, mainly the neural networks and fuzzy logic, are being applied in the estimation of battery's SoC [10, 22]. Neural networks are computationally expensive, which can overload the BMS and thus this approach, though theoretically feasible, may not be suitable for online implementation that requires instantaneous feedback and action. Also, neural networks require huge datasets in the time-consuming training process. Other techniques for SoC, include the sliding mode observer, are reported in [12].

In this work, a mathematical derivation leading to a state-space model is presented. The basic schematic model is adopted from [18, 20]. A thorough analysis in the form of state variables with the application of the Kalman filter is presented. The rest of the paper is organized as follows. The mathematical model is derived in Section 2, resulting in a state-space model. Further, in Section 3, the KF is applied to estimate the SoC of a battery system. This is followed by the tuning of KF's parameter by adopting a metaheuristic approach, namely, a genetic algorithm in Section 4. Relevant results are presented in Section 5, and finally the conclusions are derived in Section 6.

## 2. Battery Modeling

Many model-based state-estimations have been proposed in [18, 20, 21, 23]. In [18, 21], the well-known Kalman filter [16] had been applied successfully for both state observation and prediction of the SoC. Work in [24] utilized manufacturers' data in modeling the dynamic behavior of the battery. Several battery models exist from many works over the past few years. Each of these models varies in terms of its complexity and applications. In this work, a dynamical battery model is adopted, consisting of state variable equations, from [20, 21]. The schematic representation of this model is shown in Figure 2. In this model, there exists a bulk capacitor *C*_bk_ that acts as an energy storage component in the form of the charge, a capacitor that models the surface capacitance and diffusion effects within the cell *C*_surface_, a terminal resistance *R*_*t*_, a surface resistance *R*_*s*_, and an end resistance *R*_*e*_. The voltages across both capacitors are denoted as *V*_*Cb*_ and *V*_*Cs*_, respectively.

### 2.1. Mathematical Derivations of Battery Model

In this derivation, we aim to form a state-space model consisting of the state variables *V*_*Cb*_, *V*_*Cs*_, and *V*_0_. State variables are mathematical descriptions of the “state” of a dynamic system. In practice, the state of a system is used to determine its future behavior. Models that consist of a paired first-order differential equations are in the state-variable form.

Following the voltages and currents illustrated in Figure 2, the terminal voltage *V*_0_ can be expressed as

(2) $$\begin{array}{c} V_{0}=IR_{t}+I_{b}R_{e}+V_{Cb}, \end{array}$$

which is similar to

(3) $$\begin{array}{c} V_{0}=IR_{t}+I_{s}R_{s}+V_{Cs}. \end{array}$$

By (2) and (3), and following straightforward algebraic manipulation, *V*_0_ can be written as

(4) $$\begin{array}{c} I_{b}R_{e}=I_{s}R_{s}+V_{Cs}-V_{Cb}. \end{array}$$

From Kirchoff's current law, *I* = *I*_*b*_ + *I*_*s*_,

(5) $$\begin{array}{c} I_{s}=I-I_{b}. \end{array}$$

Thus, substituting (5) into (4) yields

(6) $$\begin{array}{c} I_{b}\left(R_{e}+R_{s}\right)=IR_{s}+V_{Cs}-V_{Cb}. \end{array}$$

By assuming a slow varying *C*_bk_, that is, $I_{b}=C_{\text{bk}}{\dot{V}}_{Cb}$ (from basic formula of *i* = *C*(∂*V*/∂*t*)), and then substituting it into (6), and after rearranging results in

(7) $$\begin{array}{c} {\dot{V}}_{Cb}=\frac{IR_{s}}{C_{\text{bk}}\left(R_{e}+R_{s}\right)}+\frac{V_{Cs}}{C_{\text{bk}}\left(R_{e}+R_{s}\right)}-\frac{V_{Cb}}{C_{\text{bk}}\left(R_{e}+R_{s}\right)}. \end{array}$$

By applying a similar derivation, the rate of change of the surface capacitor voltage ${\dot{V}}_{Cs}$, derived also from (2) and (3), gives

(8) V˙Cs=IReCsurface(Re+Rs)−VCsCsurface(Re+Rs) +VCbCsurface(Re+Rs).

Further, by assuming *A* = 1/*C*_bk_(*R*_*e*_ + *R*_*s*_) and *B* = 1/*C*_surface_(*R*_*e*_ + *R*_*s*_), (7) and (8) can be written as

(9) $$\begin{array}{c} {\dot{V}}_{Cb}=A\cdot I\cdot R_{s}+A\cdot V_{Cs}-A\cdot V_{Cb},\\ {\dot{V}}_{Cs}=B\cdot I\cdot R_{e}-B\cdot V_{Cs}+B\cdot V_{Cb}, \end{array}$$

respectively. Further, (9) and (10) can be combined to form a state variable relating voltages *V*_*Cs*_ and *V*_*Cb*_ and current flow *I*, which is

(10) $$\begin{array}{c} \left[\begin{array}{c} {\dot{V}}_{Cb}\\ {\dot{V}}_{Cs} \end{array}\right]=\left[\begin{array}{cc} -A & A\\ B & -B \end{array}\right]\left[\begin{array}{c} V_{Cb}\\ V_{Cs} \end{array}\right]+\left[\begin{array}{c} A\cdot R_{s}\\ B\cdot R_{e} \end{array}\right]I. \end{array}$$

Next, the output voltage is derived from (2) and (3). By adding both equations, we obtain

(11) $$\begin{array}{c} 2V_{0}=2IR_{t}+I_{b}R_{e}+I_{s}R_{s}+V_{Cb}+V_{Cs}. \end{array}$$

Then by substituting *I*_*b*_ = *R*_*s*_/(*R*_*s*_ + *R*_*e*_)  ·  *I* and *I*_*s*_ = *R*_*e*_/(*R*_*s*_ + *R*_*e*_)  ·  *I* into (11), it is further simplified as

(12) $$\begin{array}{c} V_{0}=\frac{V_{Cb}+V_{Cs}}{2}+\left(R_{t}+\frac{R_{e}R_{s}}{R_{e}+R_{s}}\right)I. \end{array}$$

Taking the time derivative of the output voltage and assuming *dI*/*dt* ≈ 0 (this simply means that the change rate of terminal current can be ignored when implemented digitally), we obtain

(13) $$\begin{array}{c} {\dot{V}}_{0}=\frac{{\dot{V}}_{Cb}+{\dot{V}}_{Cs}}{2}. \end{array}$$

Substituting the formulae obtained in (9) and (10) into (13) results in

(14) $$\begin{array}{c} 2{\dot{V}}_{0}=\left(-A+B\right)V_{Cb}+\left(A-B\right)V_{Cs}+\left(AR_{s}+BR_{e}\right)I. \end{array}$$

Then, solving for *V*_*Cs*_ from (12) we obtain

(15) $$\begin{array}{c} V_{Cs}=2V_{0}-2\left(R_{t}+\frac{R_{e}R_{s}}{R_{e}+R_{s}}\right)I-V_{Cb}, \end{array}$$

and after substituting it into (14), it yields

(16) V˙0=(−A+B)VCb+(A−B)V0 +[A(0.5Rs+Rt+D)+B(0.5Re−Rt−D)]I.

Finally, the complete state variable network is obtained by substituting (16) into (10), represented in matrix form as

(17) [V˙CbV˙CsV˙0]=[−AA0B−B0(−A+B)0(A−B)]·[VCbVCsV0] +[A·RsB·ReA(0.5Rs−Rt−D)+B(0.5Re+Rt+D)]I,

whereby constants *A*, *B*, and *D* were derived previously but are hereby restated as

(18) $$\begin{array}{c} \left[\begin{array}{c} A\\ B\\ D \end{array}\right]=\left[\begin{array}{c} \frac{1}{C_{\text{bk}}\left(R_{e}+R_{s}\right)}\\ \frac{1}{C_{\text{surface}}\left(R_{e}+R_{s}\right)}\\ \frac{R_{e}R_{s}}{R_{e}+R_{s}} \end{array}\right]. \end{array}$$

This completes the initial derivation of the battery model.

### 2.2. Numerical Example

By substituting all capacitor and resistor values from Table 1 into (18), the following values are obtained:

(19) $$\begin{array}{c} \left[\begin{array}{c} A\\ B\\ D \end{array}\right]=\left[\begin{array}{c} 0.001508759347566\\ 1.623837940973491\\ 0.001875000000000 \end{array}\right]. \end{array}$$

By defining matrix **A**,

(20) $$\begin{array}{c} A=\left[\begin{array}{ccc} -A & A & 0\\ B & -B & 0\\ \left(-A+B\right) & 0 & \left(A-B\right) \end{array}\right], \end{array}$$

(21) $$\begin{array}{c} B=\left[\begin{array}{c} A\cdot R_{s}\\ B\cdot R_{e}\\ A\left(0.5R_{s}-R_{t}-D\right)+B\left(0.5R_{e}+R_{t}+D\right) \end{array}\right]. \end{array}$$

Again by substituting the values from Table 1 to calculate *A*, *B*, and *D*, we obtain the value of **A** as

(22) $$\begin{array}{c} A=\left[\begin{array}{ccc} -1.51\;\times \;10^{-3} & 1.51\;\times \;10^{-3} & 0\\ 1.6238 & -1.6238 & 0\\ 1.6223 & 0 & -1.6223 \end{array}\right] \end{array}$$

and **B** as

(23) $$\begin{array}{c} B=\left[\begin{array}{c} 5.66\;\times \;10^{-6}\\ 6.08\;\times \;10^{-3}\\ 1.05\;\times \;10^{-2} \end{array}\right]. \end{array}$$

As such (17) can be rewritten as

(24) $$\begin{array}{c} \left[\begin{array}{c} {\dot{V}}_{Cb}\\ {\dot{V}}_{Cs}\\ {\dot{V}}_{0} \end{array}\right]=A\cdot \left[\begin{array}{c} V_{Cb}\\ V_{Cs}\\ V_{0} \end{array}\right]+B\cdot I, \end{array}$$

or numerically as

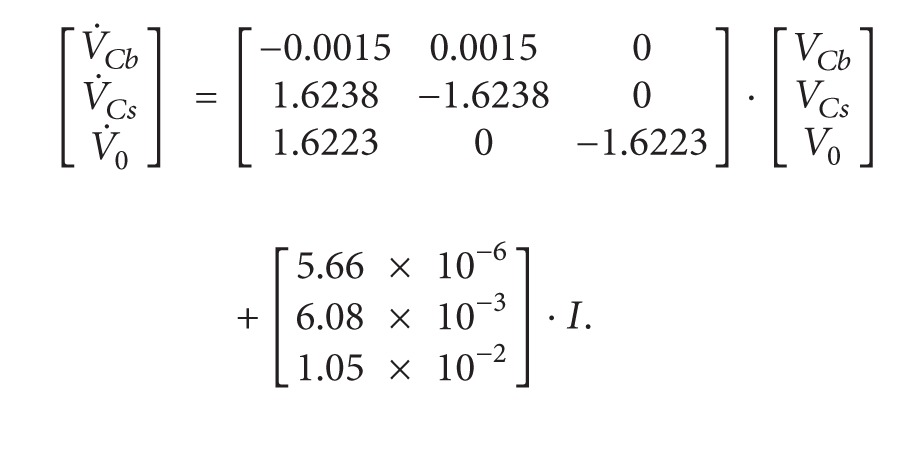
(25)


### 2.3. State-Space Modeling

Based on control theories, a lumped linear network can be written in the form

(26) $$\begin{array}{c} \dot{x}\left(t\right)=Ax\left(t\right)+Bu\left(t\right),\\ y\left(t\right)=Cx\left(t\right)+Du\left(t\right), \end{array}$$

where in this work, the state variable $\dot{x}(t)$ is

(27) $$\begin{array}{c} \dot{x}\left(t\right)=\left[\begin{array}{c} {\dot{V}}_{Cb}\\ {\dot{V}}_{Cs}\\ {\dot{V}}_{0} \end{array}\right]. \end{array}$$

Obviously,

(28) $$\begin{array}{c} x\left(t\right)=\left[\begin{array}{c} V_{Cb}\\ V_{Cs}\\ V_{0} \end{array}\right], \end{array}$$

with

(29) $$\begin{array}{c} u\left(t\right)=I. \end{array}$$

Therefore, by restating the previous calculation values in (21) and (23), we should note that the values of **A**, **B**, **C**, and **D** are as follows:

(30) A=[−1.51  ×  10−31.51  ×  10−301.6238−1.623801.62230−1.6223],

(31) B=[5.66  ×  10−66.08  ×  10−31.05  ×  10−2],

(32) $$\begin{array}{c} C=\left[\begin{array}{ccc} 0 & 0 & 1 \end{array}\right], \end{array}$$

(33) D=[0],

respectively. As such, with (32), the output *y*(*t*) is in fact

(34) $$\begin{array}{c} y\left(t\right)=V_{0}. \end{array}$$

This means that the output of the system is the open terminal voltage *V*_0_, as expected. Note that this is an important observation from this state-space modeling.

Further, the above state-space variables are transformed to a transfer function, *G*(*s*). This is done by using *ss*2*tf* function in MATLAB, which after factorization yields

(35) $$\begin{array}{c} G\left(s\right)=\frac{0.01054s^{2}+0.0171s+2.981\;\times \;10^{-5}}{s^{3}+3.248s^{2}+2.637s-1.144\;\times \;10^{-18}}. \end{array}$$

**Figure 3:**
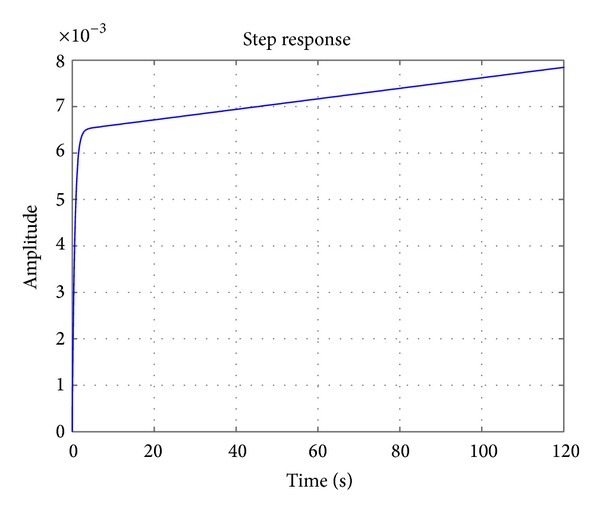
Output response of RC model due to constant input.

The plot of the unit step response for the gain in (35) is given in Figure 3. Basically, it shows that the open circuit terminal voltage *V*_0_ in Figure 2 increases linearly during the charging operation in a very slow manner after transient behaviour for few seconds.

### 2.4. Observability of the RC Battery Model

In control theory, observability is the degree to which the internal states of a system can be predicted via its external outputs. As such, for an observable system, the behavior of the entire system can be predicted via the system's outputs. On the other hand, if a system is not observable, the current values of some of its states cannot be estimated through the output signal. This means the controller does not know the states' values. In theory, the observability of a system can be determined by constructing an observability matrix *O*_*b*_.

(36) $$\begin{array}{c} O_{b}=\left[\begin{array}{cc} C & \\ CA & \\ CA^{2} & \\ \vdots & \\ CA^{n-1} & \end{array}\right], \end{array}$$

and a system is observable if the row rank of *O*_*b*_ is equal to *n* (this is also known as a full rank matrix). The ultimate rationale of such a test is that if *n* rows are linearly independent, then each of the *n* states is viewable through linear combinations of the output *y*(*t*).

Further, substituting **A** and **C** values from (30) and (32) into (36) yields

(37) $$\begin{array}{c} O_{b}=\left[\begin{array}{ccc} 0 & 0 & 1\\ 1.6223 & 0 & -1.6223\\ -2.6344 & 0.0024 & 2.6320 \end{array}\right]. \end{array}$$

Clearly, in this case *O*_*b*_ is a full rank matrix, which concludes that this system is observable.

## 3. Kalman Filter for SoC Estimation

A continuous time-invariant linear system can be described in the state variable form as

(38) $$\begin{array}{c} \dot{x}\left(t\right)=Ax\left(t\right)+Bu\left(t\right),\\ y\left(t\right)=Cx\left(t\right), \end{array}$$

where *u*(*t*) is the input vector, *x*(*t*) is the state vector, *y*(*t*) is the output vector, **A** is the time invariant dynamic matrix, **B** is the time invariant input matrix, and **C** is the time invariant measurement matrix.

If we assume that the applied input *u* is constant during each sampling interval, a discrete-time equivalent model of the system will now be

(39) $$\begin{array}{c} x\left(n+1\right)=A_{d}\cdot x\left(n\right)+B_{d}\cdot u\left(n\right),\\ y\left(n+1\right)=C_{d}\cdot x\left(n+1\right), \end{array}$$

where

(40) $$\begin{array}{c} A_{d}\approx I+A\cdot T_{c},\;\;B_{d}=B\cdot T_{c},\;\;C_{d}=C, \end{array}$$

whereby **I** is the identity matrix and *T*_*c*_ is the sampling period. As for this system, two independent process noises are present which are additive Gaussian noise, **w** vector representing system disturbances and model inaccuracies and **V** vector representing the effects of measurement noise.

Both **w** and **v** have a mean value of zero and the following covariance matrices:

(41) $$\begin{array}{c} E\left[w\cdot w^{T}\right]=Q,\\ E\left[v\cdot v^{T}\right]=R, \end{array}$$

where *E* denotes the expectation (or mean) operator and superscript *T* means the transpose of the respective vectors. In usual case, **Q** and **R** are normally set to a constant before simulation; in our case both are set to one (see Section 5). Further, a genetic algorithm (GA) is applied in order to obtain a better set of **Q** and **R** values resulting in lower RMS error from the KF's output.

**Figure 4:**
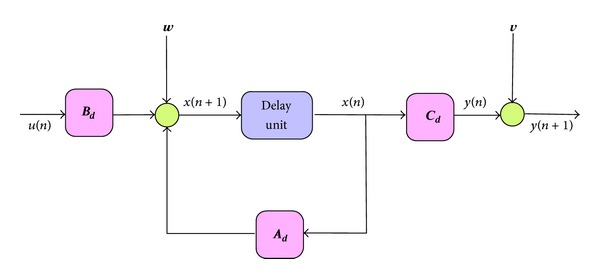
Discrete system model with noises **w** and **v**.

By inclusion of these noises, the resulting system can now be described by

(42) $$\begin{array}{c} x\left(n+1\right)=A_{d}\cdot x\left(n\right)+B_{d}\cdot u\left(n\right)+w,\\ z\left(n+1\right)=C_{d}\cdot x\left(n+1\right)+v, \end{array}$$

which is illustrated in Figure 4,

### 3.1. Property of Kalman Filter

An important property of the KF is that it minimizes the sum-of-squared errors between the actual value *x* and estimated states $\hat{x}$, given as

(43) $$\begin{array}{c} f_{\min}\left(x\right)=E\left(\left[x-\hat{x}\right]\cdot {\left[x-\hat{x}\right]}^{T}\right). \end{array}$$

To understand the operations of the KF, the meaning of the notation $\hat{x}\left(m|n\right)$ is crucial. Simply stated, it means that the estimate of *x* at event *m* takes into account all discrete events up to event *n*. As such, (43) can include such information, now expanded as

(44) $$\begin{array}{c} f_{\min}\left(x\right)=E\left(\left[x\left(n\right)-\hat{x}\left(n\;|\;n\right)\right]\cdot \left[x\left(n\right)-\hat{x}{\left(n\;|\;n\right)}^{T}\right]\right). \end{array}$$

In recursive implementation of the KF, the current estimate $\hat{x}(n|n)$, together with the input *u*(*n*) and measurement signals *z*, is used for further estimating $\hat{x}(n+1|n+1)$. This means that in this discrete system, the input for each sample step will be *u*_1_, *u*_2_, *u*_3_,…, *u*_*n*+1_ with respect to the output of *y*_1_, *y*_2_, *y*_3_,…, *y*(*n* + 1). The recursive KF algorithm is obtained with the predictor and corrector stages.

### 3.2. KF Online Implementation

For the case of a battery, it is well understood that only the terminal quantities can be measured (terminal voltage *V*_0_ and current *I*). Assuming that battery parameters are time-invariant quantities, the recursive KF algorithm is applied. By applying (40) into (30)–(32), we obtain the following values of updated matrices, with *T*_*c*_ = 1:

(45) Ad=[0.99841.51  ×  10−301.62380.623801.622300.6223],Bd=[5.66  ×  10−66.08  ×  10−31.05  ×  10−2],Cd=[001].

**Figure 5:**
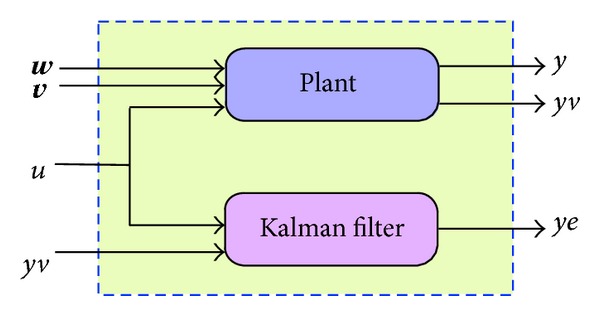
Parallel connection of plant and Kalman filter.

Note that **B**_**d**_ and **C**_**d**_ remain similar to their previous values, as given in (31) and (32). By utilizing MATLAB's control toolbox, the KF is placed in parallel to the state-space model, hereby represented by plant in Figure 5. The complete source code is given in the Appendix.

## 4. Genetic Algorithm

The genetic algorithm (GA), introduced by John Holland, is an approach based on biological evolution [25]. The algorithm is developed based on Charles Darwin's theory of survival of the fittest. The GA has a very powerful encoding mechanism that enables the representation of a solution vector as either a real coded or binary string. Both encodings serve a different purpose in the context of different problem space. GAs are regarded as the global optimizer that often spot or locate the potential area or even accurately obtain the best solution, known as the global minimum [26–28]. Underneath this popular algorithm are the three operators that contribute to its success in performing optimization task.

- *Selection*. In selection, offsprings with higher fitness have better chance for survival to the following generation in the evolutionary process. This basically is based on the theory of “survival of the fittest.”
- *Crossover*. The crossover increases and maintains the diversity of the entire population over the entire run. This is due to the fact that a population with higher diversity has the ability to explore a wider range of search space.
- *Mutation*. This enables chromosomes (potential solutions) to jump to a wider range than crossover. Again, mutation also increases the diversity of the entire population.

**Figure 6:**
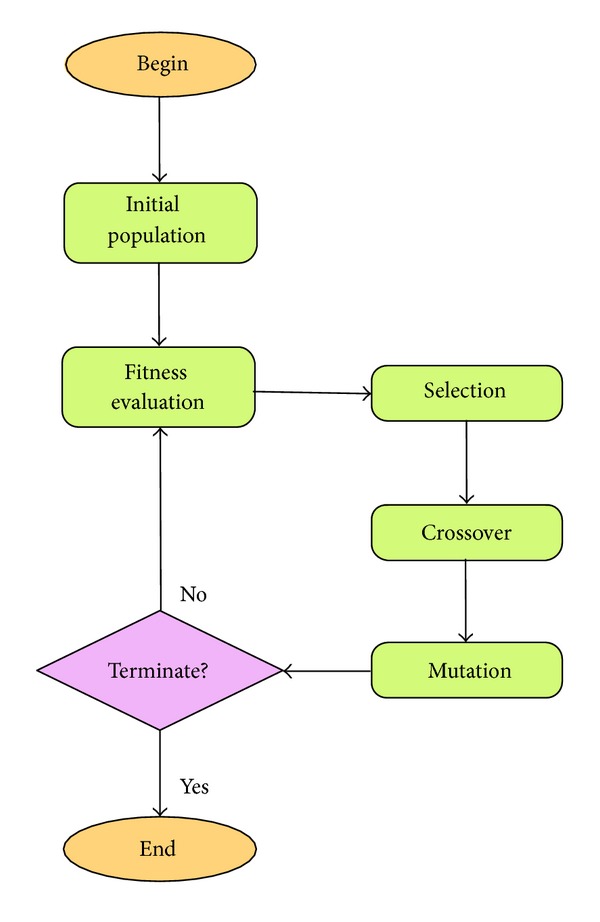
GA flowchart.

The pseudocode of the GA is presented in Algorithm 1, and relevant figure depicting the algorithm flow is illustrated in Figure 6. To avoid extended discussions on GA, we include here a complete workable source code in the appendix. All parameter settings for the GA are available in the source code.

In the context of Kalman filter, GA is applied to tune the **Q** and **R** parameters, which was explained in detail in Section 3.

## 5. Results

**Figure 7:**
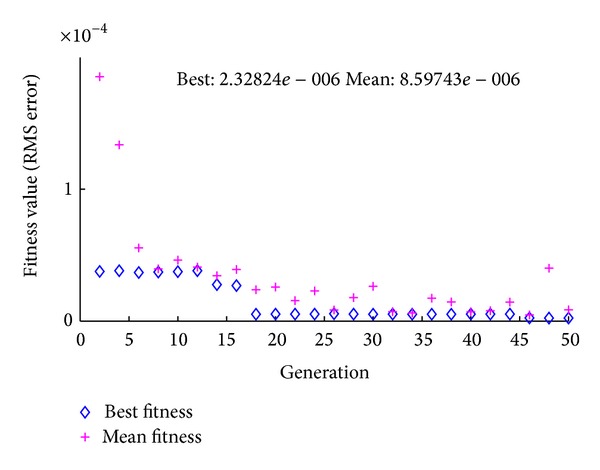
The convergence characteristic of GA.

The program, implemented in MATLAB, is given in the appendix to clarify the results obtained in this work. Take note that the **Q** and **R** mentioned in (41) are both set to a numerical value of one (**Q** = **R** = 1) in the first simulation. The results obtained are tabulated in Table 2. From these results, RMS of the estimated error, which is the error from KF, is far smaller in comparison to the measured error, with values of 1.0013 V and 1.92 × 10^−4^ V, respectively. This RMS error is further minimized by utilizing **Q** = 0.012697316315642 and **R** = 2.303940992875865, found using the GA approach. A graph depicting the convergence characteristic is shown in Figure 7.

**Figure 8:**
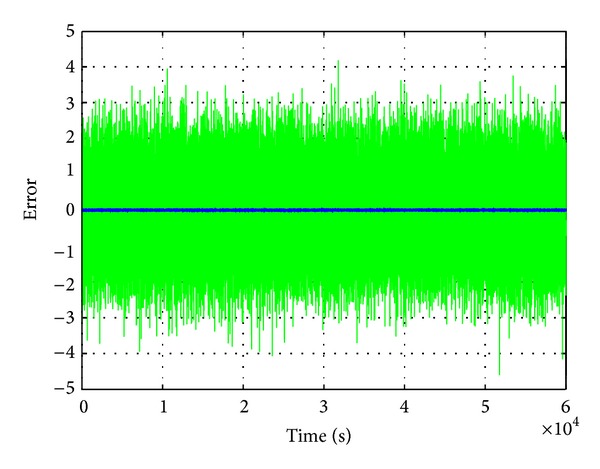
The voltage error recorded and depicting measured (green color) and estimated (blue color) errors.

The time plot of the estimated error from 0 s to 60000 s is shown in Figure 8, depicting a very small amplitude, in blue color (≈±  0.04 V) along the timeline. This is observed through the zoomed display of the MATLAB graph. On the contrary, the measurement error, portrayed by green color noise in Figure 8, has an absolute magnitude of ≈±  2 V.

### 5.1. Charging Behaviour

**Figure 9:**
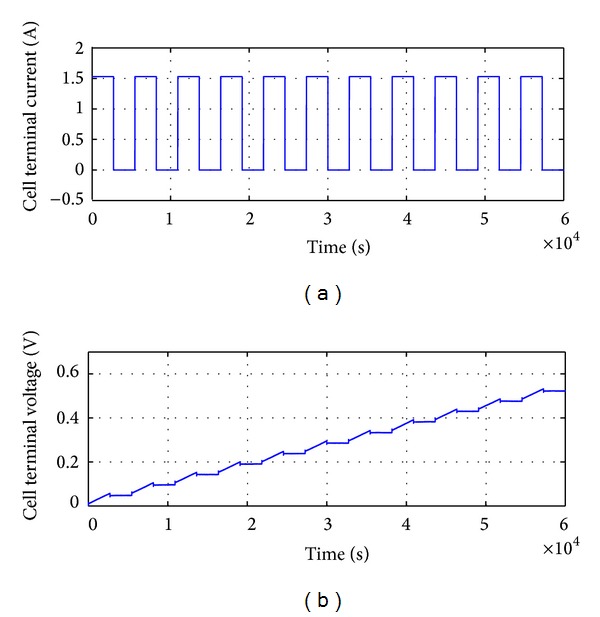
Response of RC battery model in terms of *V*_0_ due to charging current *I* = 1.53 A pulses.

The charging characteristic is illustrated in Figure 9 whereby the initial terminal voltage *V*_0_ starts from 0 V and rises up to approximately 0.5 V within 60000 seconds (which is 100 minutes). Charging impulses of 1.53 A are applied in this case as shown in Figure 9. As expected, this is a time consuming process as in general case it may take hours for a car battery (lead acid) to be completely charged.

### 5.2. Discharging

**Figure 10:**
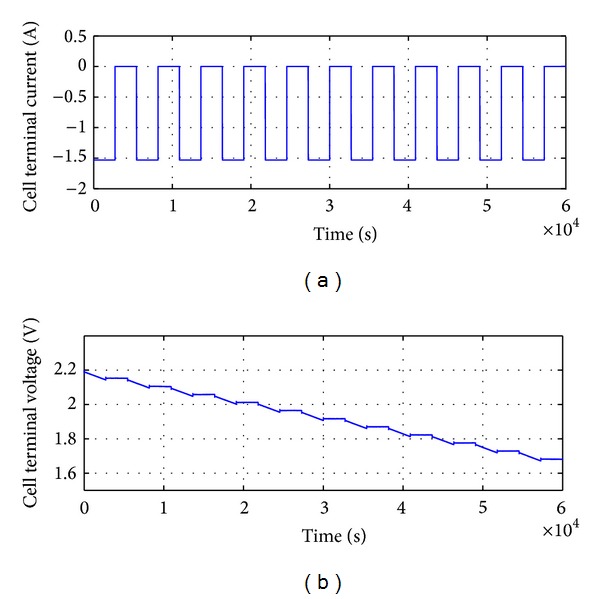
Response of RC battery model in terms of *V*_0_ due to discharge current *I* = −1.53 A pulses.

For the discharging process, the initial value of terminal voltage, *y*_0_ = *V*_0_, is set to 2.2 V in the MATLAB program. Again, in this case, impulses of 1.53 A are applied, but now in negative form. The dynamic behavior of the discharging characteristic is shown in Figure 10. From this figure, it is observed that the discharging process is similar to the charging process, but now with a linearly decreasing terminal voltage slope. The open terminal voltage *V*_0_ drops from 2.2 V to 1.7 V in 60000 seconds (100 minutes); this is similar to the charging process as it takes 100 minutes to reach *V*_0_ = 0.5 V from zero potential.

## 6. Conclusion

In this work, we successfully obtained the state variables of the RC model representing a battery in terms of mathematical derivations. The derivations lead to the conclusion that there exist three state variables relevant to a battery's state-space model. With this state-estimation model, a prominent technique known as the Kalman filter is applied in the aim of estimating state-of-charge for a Battery Management System. From numerical results, the KF is shown to be accurate in predicting the dynamic behavior of the system. This is shown by a very small RMS error of the estimation in comparison to its measurement. The estimated error is further reduced after incorporating the optimized values of **Q** and **R** through the offline GA approach. As such, the efficacy of such an approach is, thus, validated.

## Figures and Tables

**Algorithm 1:**
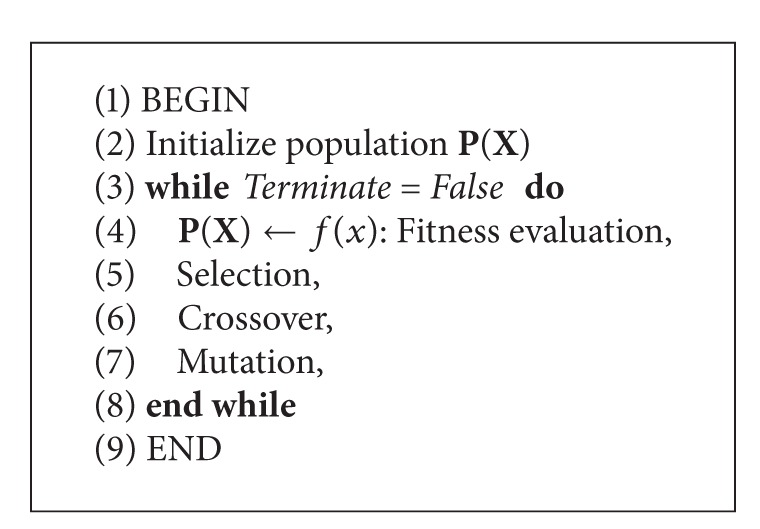
Pseudocode of GA.

**Table 1:** Parameters for cell model [18, 20].

*C*_bk_	*C*_surface_	*R*_*e*_	*R*_*s*_	*R*_*t*_
88372.83 F	82.11 F	0.00375 Ω	0.00375 Ω	0.002745 Ω

**Table 2:** RMS error recorded during charging operation.

Output	**Q**, **R**	RMS error [V]
Measurement, *y* − *y*_*v*_	1, 1	1.0013

Estimated (KF), *y* − *y*_*e*_	1, 1	1.9185 × 10^−4^

Estimated (KF), *y* − *y*_*e*_	0.012697316315642,	2.3282 × 10^−6^
(After GA tuning)	2.303940992875865	

## Appendix

### A. MATLAB Source Code, in a Script File

format long;

randn(‘seed',0)  %Same sequence

%Value for resistors and capacitors

Csurface=82.11;

Cbk=88372.83;

Re=0.00375;

Rs=0.00375;

Rt=0.002745;

a=1/(Cbk∗(Re+Rs));

b=1/(Csurface∗(Re+Rs));

d=(Re∗Rs)/(Re+Rs);

%State variable matrices

A=[−a a 0; b −b 0; (−a+b) 0 (a−b) ];

B=[a∗Re; b∗Re;

  a∗ (0.5∗Rs−Rt−d)+ b∗(0.5∗Re+Rt+d)];

C=[0 0 1 ];

D=[0];

%Transfer function

figure(1); %Figure  1

[num, den]=ss2tf(A,B,C,D,1);

G=tf(num,den)

step(G),grid;

% For Kalman filter:

% Identity matrix + diagonal element

A=[1−a a 0; b 1−b 0; (−a+b) 0 1+(a−b) ]

B=[a∗Re; b∗Re;

   a∗ (0.5∗Rs−Rt−d)+ b∗(0.5∗Re+Rt+d)]

C=[  0 0 1  ]

Tc=1;

A=A∗Tc;

B=B∗Tc;

C=C;

x=[2,2]

options = gaoptimset(…

‘PopulationType', ‘doubleVector',…

‘PopInitRange', [0;1],…

‘PopulationSize', 5,…

‘EliteCount', 1,…

‘CrossoverFraction', 0.8,…

‘ParetoFraction', 0.35,…

‘MigrationDirection', ‘both',…

‘MigrationInterval', 20,…

‘MigrationFraction', 0.2,…

‘Generations', 50,…

‘TimeLimit', Inf,…

‘FitnessLimit', −Inf,…

‘StallGenLimit', 50,…

‘StallTimeLimit', Inf,…

‘TolFun', 0,…

‘TolCon', 0,…%1e−6,…

‘InitialPopulation', x,…

‘InitialScores', [],…

‘InitialPenalty', 10,…

‘PenaltyFactor', 100,…

‘CreationFcn', @gacreationuniform,…

‘FitnessScalingFcn', @fitscalingrank,…

‘SelectionFcn', @selectionroulette,…

‘CrossoverFcn', @crossoverarithmetic,…

‘MutationFcn', @mutationadaptfeasible,…

‘DistanceMeasureFcn',  @distancecrowding,…

‘HybridFcn', [],…

‘Display', ‘final',…

‘OutputFcns', [],…

‘PlotFcns', @gaplotbestf,…

‘PlotInterval', 2,…

‘Vectorized', ‘off',…

‘UseParallel', ‘always');

Fmin =@(x)fobj(x, A, B, C);

lb=[0.001,0.001]; %Set lower bounds

v[X,fval,exitflag ]  =…

ga(Fmin, 2, [],[],[],…

    [], lb, [],[], options );

% After simulation,

% repeat simulation all plot all graphs

if fval<0.0001

Q=X(1); R=X(2);

% Sample time=−1 for discrete model

Plant = ss(A,[B B],C,0,−1,…

‘inputname',{‘u'  ‘w'},…

‘outputname', ‘y');

[kalmf,L,P,M]  = kalman(Plant,Q,R);

kalmf = kalmf(1,:);

Kalmf

a = A;

b = [B B 0∗B];

c = [C;C];

d = [0 0 0;0 0 1];

P = ss(a,b,c,d,−1,…

‘inputname',{‘u'   ‘w'  ‘v'},…

‘outputname',{‘y'   ‘yv'});

sys = parallel(P,kalmf,1,1,[],[])

% Close loop for input #4 and output #2

SimModel = feedback(sys,1,4,2,1)

% Delete yv from I/O list

SimModel = SimModel([1 3],[1 2 3])

SimModel.inputname

%This part generates rectangular waveform

A=1.53; %Amplitude of negative pulses

t = 0:1:60000;

T=30000/5.5;

% I here represents current

I = (A/2)∗square(2∗pi∗(1/T)∗t);

I = I+(A/2);

figure(1); %Figure  2

subplot(211),plot(t,I),

axis([0 60000 −0.5 2.0]);

grid on; %charging

xlabel(‘Time (sec)');

ylabel(‘Cell terminal current (A)');

u = I';

n = length(t);

%randn(‘seed',0)  %Same sequence

w = sqrt(Q)∗randn(n,1);

v = sqrt(R)∗randn(n,1);

[out,x] = lsim(SimModel,[w,v,u]);

y0=0; %This is initial terminal voltage

y = out(:,1)+y0;    % true response

ye = out(:,2)+y0;   % filtered response

yv = y + v;    % measured response

figure(2);

plot(t,y, ‘g−−',t,ye, ‘b−'), grid on;

figure(1);

subplot(212),plot(ye, ‘b−'),

axis([0 60000 0 0.7]); grid on; %charging

xlabel(‘Time (s)'),

ylabel(‘Cell terminal voltage (V)')

%Kalman filter response

figure(3); %Figure  3

plot(t,y−yv, ‘g−', t, y−ye, ‘b−'), grid on;

xlabel(‘Time (s)'), ylabel(‘Error')

%Calculate Errors

MeasErr = y−yv;  %Measurement error

MeasErrCov=...

sum(MeasErr.∗MeasErr)/length(MeasErr);

EstErr = y−ye;  %Estimated error

EstErrCov =…

sum(EstErr.∗EstErr)/length(EstErr);

%Display onto screen

MeasErrCov  %Measurement error

EstErrCov  %Estimated error

End

### B. MATLAB Source Code, in a Function File

function f = fobj(x, A, B, C)

Q=x(1);

R=x(2);

% Sample time=−1 for discrete model

Plant = ss(A,[B B],C,0,−1,…

   ‘inputname',{‘u'  'w'},…

   ‘outputname',  ‘y');

[kalmf,L,P,M]  = kalman(Plant,Q,R);

kalmf = kalmf(1,:);

Kalmf

a = A;

b = [B B 0∗B];

c = [C;C];

d = [0 0 0;0 0 1];

P = ss(a,b,c,d,−1,…

   ‘inputname',{‘u'  ‘w'  ‘v'},…

   ‘outputname',{‘y'  ‘yv'});

sys = parallel(P,kalmf,1,1,[],[])

% Close loop around input #4 and output #2

SimModel = feedback(sys,1,4,2,1)

% Delete yv from I/O list

SimModel = SimModel([1 3],[1 2 3])

SimModel.inputname

%This part generates rectangular waveform

A=1.53; %Amplitude of negative pulses

t = 0:1:60000;

T=30000/5.5;

% I here represents current

I = (A/2)∗square(2∗pi∗(1/T)∗t);

I = I+(A/2);

u = I';

n = length(t);

%randn(‘seed',0)  %Same sequence

w = sqrt(Q)∗randn(n,1);

v = sqrt(R)∗randn(n,1);

[out,x]  = lsim(SimModel,[w,v,u]);

y0=0; %This is initial terminal voltage

y = out(:,1)+y0;  % true response,

ye = out(:,2)+y0;   % filtered response

yv = y + v;     % measured response

%Calculate Errors

MeasErr = y−yv;  %Measurement error

MeasErrCov= …

sum(MeasErr.∗MeasErr)/length(MeasErr);

EstErr = y−ye;  %Estimated error

EstErrCov = …

sum(EstErr.∗EstErr)/length(EstErr);

f=EstErrCov;

end
